# Evolution of a predator-induced, nonlinear reaction norm

**DOI:** 10.1098/rspb.2017.0859

**Published:** 2017-08-23

**Authors:** Mauricio J. Carter, Martin I. Lind, Stuart R. Dennis, William Hentley, Andrew P. Beckerman

**Affiliations:** 1 Centro Nacional del Medio Ambiente, Universidad de Chile, Avenida Larrain 9975, La Reina, Santiago, Chile; 2 Departamento de Ecología, Facultad de Ecología y Recursos Naturales, Universidad Andres Bello, Santiago, Chile; 3 Animal Ecology, Department of Ecology and Genetics, Uppsala University, Uppsala 75236, Sweden; 4 Department of Aquatic Ecology, Eawag, Überlandstrasse 133, 8600 Dübendorf, Switzerland; 5 Department of Animal and Plant Sciences, University of Sheffield, Western Bank, Sheffield S10 2TN, UK

**Keywords:** reaction norm, evolution, predator-induced plasticity, morphological defence, *Daphnia pulex*

## Abstract

Inducible, anti-predator traits are a classic example of phenotypic plasticity. Their evolutionary dynamics depend on their genetic basis, the historical pattern of predation risk that populations have experienced and current selection gradients. When populations experience predators with contrasting hunting strategies and size preferences, theory suggests contrasting micro-evolutionary responses to selection. *Daphnia pulex* is an ideal species to explore the micro-evolutionary response of anti-predator traits because they face heterogeneous predation regimes, sometimes experiencing only invertebrate midge predators and other times experiencing vertebrate fish and invertebrate midge predators. We explored plausible patterns of adaptive evolution of a predator-induced morphological reaction norm. We combined estimates of selection gradients that characterize the various habitats that *D. pulex* experiences with detail on the quantitative genetic architecture of inducible morphological defences. Our data reveal a fine scale description of daphnid defensive reaction norms, and a strong covariance between the sensitivity to cues and the maximum response to cues. By analysing the response of the reaction norm to plausible, predator-specific selection gradients, we show how in the context of this covariance, micro-evolution may be more uniform than predicted from size-selective predation theory. Our results show how covariance between the sensitivity to cues and the maximum response to cues for morphological defence can shape the evolutionary trajectory of predator-induced defences in *D. pulex*.

## 1. Introduction

Predator-induced defences remain one of the core examples of phenotypic plasticity in the wild. Examples include dramatic changes in morphology, large- and small-scale shifts in habitat use and foraging, and change to the magnitude and direction of several life-history traits [1–8]. Several decades of research in aquatic communities show that plasticity in all these types of traits can be mobilized by exposure to predation risk cues—typically chemicals released by predators [8–17].

One of the most studied examples of adaptive phenotypic plasticity is the predator-induced morphological defence in waterfleas facing predation risk by fish or midge larvae [2,18,19]. *Daphnia pulex* is a species that faces predation risk from at least two size-selective predators: midge larvae and fish. Midge predation is typically small size-selective and results in morphological defences—called neckteeth—at the 2nd/3rd juvenile instar, the size at which they are most at risk. This is accompanied by delayed maturation at a larger size representing investment into growth over reproduction. By contrast, fish predation is typically large size-selective and results in no morphological defence in *D. pulex*, but accelerated maturation at a smaller size, indicative of investment into reproduction over growth [20]. The induced morphological defence in *D. pulex* is considered adaptive, conferring a 30–50% increase in survival [9,21].

Historically, this predator-induced morphological defence has been classified as a threshold or binary trait [22,23]. However, recent theory [24] and empirical data [8,15,19,25,26] suggest that the morphological defence can be effectively characterized by a continuous, nonlinear function, typically sigmoid, of predation risk [8,25]. In fact, given this functional form, it is possible to characterize population patterns of, and genetic variation in, the induced defence in terms of the parameters of a three-parameter sigmoid model [8,25,27]: the asymptote represents the maximum amount of defence, the inflection point the ‘threshold’ or sensitivity to predation cues, and the slope or scale parameter the ‘reactivity’, or how binary the response is. The reaction norm—how the defence varies with increasing predation risk—can be captured by three variables.

Such a characterization allows for a unique game, where we can ask how inducible defences, and the reaction norm that describes them, might evolve. Theory and data suggest that the evolution of a defence characterized by, for example, three traits, will be driven by a combination of how selection pressures target the traits, the heritability of the traits (genetic variation) and correlations among traits (trade-offs and constraints) [6–8]. Here we predict plausible patterns of micro-evolution of the *D. pulex* predator-induced morphological defence, specifically revealing how genetic (co)variation among the amount of defence, the sensitivity to the cue and the reactivity to the cue might constrain such a response. We do this by integrating field-based estimates of genetic variation in the three traits (asymptote, threshold, reactivity) with various, plausible selection regimes via the multivariate breeder's equation. Via these data and the breeders equation, we evaluate the potential micro-evolution of morphological defence reaction norms.

Our approach to make quantitative predictions about the magnitude and direction of the possible responses to selection involves four steps. First, we characterize the reaction norms for morphological predator defence as a sigmoid, three-parameter (trait) reaction norm. Second, we estimate the *G*-matrix that defines a plausible empirical sample of the variance and covariance among the three traits in nature. Third, we derive a set of five plausible selection gradients that combine selection gradient analysis of reproduction via traditional multiple regression tools [28,29] with additional information on survival drawn from size-selective predation theory. Together these data form a data platform on which we can explore potential micro-evolutionary change in reaction norms. To this end, we then combine the *G*-matrix and selection gradients via the multivariate breeder's equation to make predictions about, and visualize, plausible responses to selection by the nonlinear reaction norm of inducible morphological defence.

## 2. Material and methods

### (a) Study system

We characterized the neckteeth reaction norms of and genetic covariance matrix formed by 12 iso-female lineages of *D. pulex* [25], a keystone herbivore of algae in ponds and lakes [30]. In the UK, *D. pulex* can be subject to contrasting and seasonal predation pressure by fish in spring, and midge larvae during summer and autumn [8]. The iso-female clones were originally collected from two very different shallow source ponds in Sheffield, UK, separated by approximately 8 km [25]. Bagshaw pond (seven genotypes; 53°20′5.37″ N, 1°27′8.12″ W) contains only invertebrate predators, primarily *Chaoborus* sp. (onwards: midge pond). Crabtree pond (five genotypes; 53°24′17.46″ N, 1°27′26.81″ W) contains both fish and midge predators (onwards: fish–midge pond). Genetic differences between iso-female lines were confirmed by microsatellite analysis [31].

### (b) Reaction norms

We experimentally determined the predator-induced morphological reaction norm in controlled temperature rooms at 21°C on a 16 L : 8 D cycle. We estimated the reaction norms of the neckteeth by exposing replicate daphnids of each genotype to a gradient of chemical kairomone cues from *Chaoborus flavicans* [8,25]. We extracted kairomone from frozen *C. flavicans*, (Honka, Germany) [8,9,25]. Third-generation mothers of each genotype, at their second brood, were exposed to each concentration of chemical cue (to avoid maternal and grand-maternal effects). Five neonates (third brood, third generation) from each of three mothers, for a total of 15 neonates per genotype, were distributed individually into glass jars containing 50 ml of hard artificial pond water [32], food (*Chlorella vulgaris*; 2 × 10^5^ cells ml^−1^) and the appropriate volume of purified cue.

We defined the reaction norm along a gradient of seven concentrations of extracted predator cue (0, 0.1, 0.25, 0.5, 0.75, 1, 2 µl ml^−1^), with the exception of clone ‘carlos’ that was evaluated at four more concentrations (0.15, 0.4, 0.6, 0.8 µl ml^−1^). Media were replaced daily and induction of neckteeth was scored following established methods. Each individual was examined under the microscope daily for evidence of induction, from birth to maturation. Points were assigned to the presence of a pedestal and/or the presence of spikes. Pedestals were classified as absent (score = 0), small (score = 30) and large (score = 50). Spikes were assigned 10 points each. For individuals exhibiting neckteeth in multiple instars, we used the maximal amount of induction in analyses, regardless of the instar in which it was observed. Typically, this was either 2nd or 3rd instar. Our maximum level of induction among all replicates was 130 points (large pedestal, eight spikes). All individual scores were normalized to this maximum defining induction levels between 0 and 100 [8,19,25].

We characterized the reaction norms of neckteeth induction for each maternal replicate of each genotype by a sigmoid curve where the asymptote corresponds to maximum induction (maximum), the inflection to the concentration of cue where 50% induction occurs (threshold or sensitivity) and the slope (reactivity), corresponding to the rate of induction or how binary the reaction norm looks [8,25]. These data were estimated for each maternal replicate (i.e. three mothers; *n* = 5 reps/mother × 7 treatments) via nonlinear least-squares with a three-parameter logistic model. Parameters were estimated using the nls function and the associated three-parameter logistic model in R [33]. This analysis provided discrete and replicated trait data for each genotype (3 maternal lines × 12 clones = 36 estimates of maximum, sensitivity and reactivity), along the experimental gradient.

### (c) Fitness proxy: reproduction

The experimental exposure of all replicates to predation cues continued until all animals had reached maturity. Maturation was classified as the appearance of eggs in the brood pouch. We defined a proxy of fitness here as a function of age at first reproduction and clutch size and used this in the selection gradient analysis below:
2.1
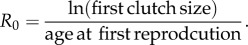


This measure of fitness is focused on reproduction and is highly correlated with population growth rate in exponentially growing populations [34]. Furthermore, our own data confirm that first clutch reproduction is highly correlated with reproduction after three clutches (*r* = 0.77, *p* < 0.001; MI Lind, K Yarlett, AP Beckerman 2014, unpublished data).

### (d) *G*-matrix of neckteeth phenotypic plasticity

We used the 36 estimates of each of the three traits among the genotypes, structured by maternal identity, to characterize a *G*-matrix. We fit a trivariate model with mother (*n* = 12) as the random effect, using Bayesian Monte Carlo Markov chain (MCMC) generalized linear mixed models (*MCMCglmm* package in R v. 3.3.1 [35]) to obtain the (broad-sense) genetic covariance matrix. We used informative, parameter expanded priors, 180 000 iterations, a burn-in period 45 000 and thinning interval 200 to estimate a joint posterior distribution of *n* = 1000. The chains were well mixed; time-series plots showed no sign of autocorrelation. This produced a modal estimate of variance and covariance among traits and allows direct estimates of broad-sense heritability and genetic correlations. Significance of genetic parameters was assessed using 95% credible intervals calculated from the joint posterior distribution [35]. We estimated the *G*-matrix as the posterior mode of the joint posterior of the variance covariance matrix.

We fit two additional models. First, we removed the random effect. The deviance information criteria (DIC) confirmed that fitting the random component estimated a substantial amount of variation. Second, we tested the significance of covariance structure by comparing a model setting the off diagonal elements of the *G*-matrix to zero using the ‘idh()’ function instead of ‘us()’ function for the random effect (see *MCMCglmm* package in *R* v. 3.3.1 [35]). Again, a DIC comparison indicated significant covariance.

### (e) Defining selection gradients

Our approach for estimating selection gradients follows [27]. Selection under predation risk in nature depends upon both reproduction and survival. Their relative importance may depend upon the predation regime and how predation risk varies over a season [35]. We, therefore, defined five plausible composite selection gradients, each representing a different weighting of reproduction (*β*_R_) and survival (*β*_S_): *β*_R_, *β*_R_ + 0.5*β*_S_, *β*_R_ + *β*_S_, 0.5*β*_R_ + *β*_S_ and *β*_S_ (also see Lind *et al.* [27]).

We defined the selection gradient on reproduction (*β*_R_) by regressing our fitness proxy (equation (2.1)) against the three traits defining the reaction norm [28,36]. We fit the selection gradient (fitness proxy as a function of the maximum (asymptote), sensitivity (inflection) and reactivity (scale)) as a response surface using the *rsm* package in *R* [37], which provided estimates of the linear (*β;* directional selection) and quadratic (*γ*; indirect selection) components. As has been suggested in the literature [38,39], we used a randomization test of significance for all parameters of the selection gradients. We re-estimated selection gradients from new regressions based on random allocations of fitness to predictor variables [40]. From this, we derived two-tailed probabilities of estimating selection gradients of the observed magnitudes by chance. We performed separate randomization tests of linear and nonlinear gradients in each environment

We doubled the nonlinear coefficient of multiple regression (following Stinchcombe *et al*. [41]) to standardize their magnitude with the linear and correlational gradients estimated above, which helps to avoid underestimation of disruptive or stabilizing selection. We then explored the extent of nonlinear selection by performing a canonical correspondence analysis. This method helps to identify the major axes of the overall response surface [42]. We used randomization to test the significance of all parameters of *β*_R_*,* taking the potential non-independence of residual into account [38].

The selection gradients on survival, *β*_S_ were defined from the empirical and theoretical literature of size-selective predation on *Daphnia*. This literature (see [20] for theory) is strongly focused around assumptions that gape-limited predators, such as *Chaoborous* larvae, which are able to attack small prey [43,44], leading to conspicuous defences in *D. pulex* [14,26], whereas visually hunting predators such as fish target large prey [44,45] and do not favour induced morphological defences in *D. pulex*. To reflect this, we define the *β*_S_ by how each reaction norm parameter would respond in each predation context. For example, we assume that survival is increased under small size-selective midge predation by increasing the asymptote. Table 1 provides an overview of how we characterized adaptive strategies associated with each predation regime, defined by the three variables.

**Table 1.** Hypothetical output of response of selection in *D. pulex* two predator environments with different scenarios of survival selection gradients on neckteeth reaction norm parameters.

	parameters of neckteeth induction		
	maximum	threshold	steepness
adaptive strategies	high *β*: 1	low *β*: −1	high *β*: 1
midge pond	higher expression of morphological response, survival benefit	lower sensitivity morphological response, less response costs	faster change
adaptive strategies	low *β*: −1	high *β*: 1	low *β*: 0
fish–midge pond	lower expression of morphological response, less energetic cost	higher sensitivity morphological response, higher response cost	slower change

Finally, we created the composite selection gradients by standardizing *β*_R_ and *β*_S_ to a total length (strength of selection) of 1 and producing the several combinations of reproduction (*β*_R_) and survival (*β*_S_) defined above. Each composite *β* was standardized to a length of 1 to enable meaningful comparison of the response to selection in the following step (electronic supplementary material, table S1).

### (f) The evolution of a reaction norm of inducible defence

We applied the multivariate breeders equation to the phenotypic reaction norm for each of midge and fish–midge population, demonstrating how the *G*-matrix we estimated, along with five different weightings of selection on reproduction and survival, might drive evolution of each reaction norm.

The total response to selection for each component of the reaction norm can be decomposed into direct and correlated (indirect) selection responses [46]:
2.2*a,b*
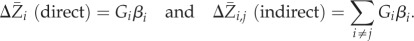


We examined whether any changes in genetic (co)variation can be ascribed to nonlinear and correlated selection (*γ*—the matrix of nonlinear selection gradient) [36].

## 3. Results

### (a) Reaction norms

**Figure 1.**
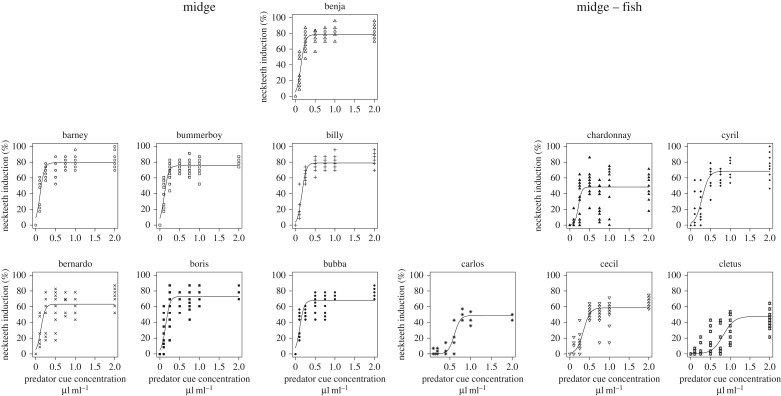
Neckteeth reaction norms of 12 *Daphnia pulex* clones facing a gradient of *Chaoborus* predation risk. Each line corresponds to a clone-specific sigmoid fit that clone's data. The data points correspond to individual replicates of each clone in each experimental condition. We fit a three-parameter sigmoid model where the asymptote is the maximum induction, the inflection is the cue concentration at which 50% induction is reached and the slope or scale is the reactivity or how the binary is the response.

**Figure 2.**
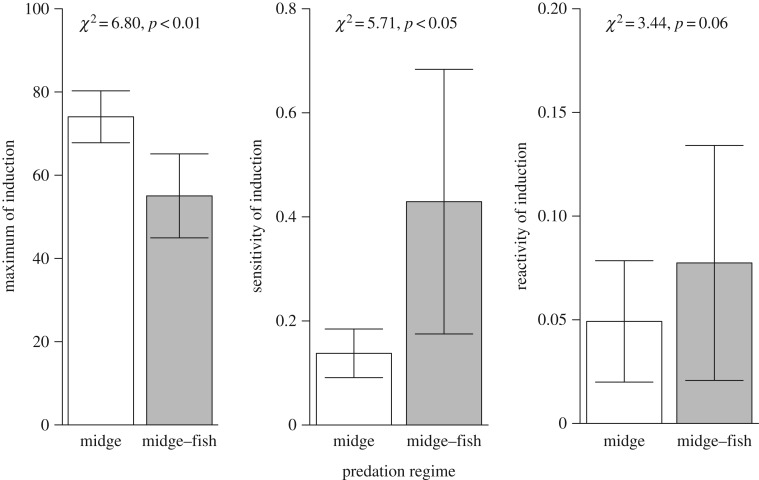
Parameter mean ± s.e. values that describe the neckteeth reaction norms to Bagshaw (midge) and Crabtree (fish–midge) populations of *Daphnia pulex*. Statistical comparison was performed with maximum likelihood analysis treating predation regime as fixed effect.

We found significant genetic and population specific variation in the maximum (asymptote) and sensitivity (threshold/inflection) of the reaction norms (figures 1 and 2). Genotypes sourced from the midge pond (Bagshaw) were characterized by high levels of induction (74.0 ± 6.24 induction) and a sensitive (closer to 0) threshold (0.13 ± 0.04 µl ml^−1^). Genotypes sourced from the fish–midge pond (Crabtree) show comparatively lower levels of induction (55.0 ± 10.1 induction) and a less sensitive threshold (0.42 ± 0.25 µl ml^−1^). A small-scale parameter (reactivity) in both ponds suggests a steep, threshold-like response (midge: 0.05 ± 0.02; fish: 0.08 ± 0.05). Our fitness proxy, defined as the log quotient of age at maturity and first clutch size, did not differ between ponds (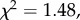 *p* = 0.22).

### (b) *G*-matrix

We found significant genetic variation in the maximum amount of induction (asymptote) and the sensitivity (threshold/inflection) to cues (table 2), with genetic variation in the sensitivity being lower than in the maximum. We also found a negative and significant genetic covariance between the maximum and sensitivity of induction (table 2). This represents a positive covariance in ‘biological’ terms as reducing the sensitivity (more sensitive to kairomones) and increasing the maximum response are both deemed ‘beneficial’ in the face of midge predation. Thus, this *G*-matrix, comprised of individuals from two populations, reflects a potentially strong constraint where the maximum induction (asymptote) and the sensitivity to predator cues (threshold/inflection) respond together in a biologically meaningful manner.

**Table 2.** G and P (co)variance matrices estimates from the pooled population (midge/fish–midge as fixed effect). The parameters correspond to mode of posterior distribution from data that were standardized to mean = 0 and s.d. = 1 prior to estimating variance components. The significant genetic parameters are indicated by (*), after comparing DIC (electronic supplementary material, table S2) between models.

	maximum (IC)	sensitivity (IC)	reactivity (IC)
*G-matrix*			
maximum	0.18 (0.03; 0.53)*	−0.12 (−1.54; −0.18)*	−0.001 (−0.72; 0.09)
sensitivity		0.35 (0.12; 0.95)*	0.03 (−0.09; 0.79)
reactivity			0.02 (0.005; 0.43)
*P-matrix*			
maximum	0.36 (0.23; 0.61)	−0.13 (−0.28; 0.45)	−0.07 (−0.30; 0.12)
sensitivity		0.53 (0.32; 0.85)	0.20 (0.03; 0.53)
reactivity			0.79(0.53; 1.36)

### (c) Selection gradients

The selection gradient estimates for our fitness proxy in each predation regime were distinct. Midge pond data revealed directional (linear) and nonlinear (disruptive) selection on maximum induction (table 3). Fish–midge pond data revealed nonlinear (disruptive) selection on the sensitivity of induction (table 3).

**Table 3.** Summary of linear and quadratic selection analyses and *M* matrix of eigenvectors from canonical analysis of *γ*_matrix_ for reaction norms parameters of both populations. The coefficients were obtained from parameters (maximum, sensitivity and reactivity) that describe the reaction norm of sigmoid fit of neckteeth *D. pulex*. Standardized directional selection coefficient (*β*), standardized nonlinear and correlated selection coefficients (*γ*_matrix_). The nonlinear coefficients reported were doubled from the originals (in parenthesis) following the suggestion of Stinchcombe *et al.* [42]. Significant coefficients were obtained by randomization (10 000) test.

		*γ*				*M* matrix		
traits	*β*	maximum	sensitivity	reactivity	*λ*_i_	maximum	sensitivity	reactivity
*midge* (*Bagshaw*)								
maximum	0.029	0.189 (0.09)*			0.227	0.581	0.779	0.231
sensitivity	0.016*	−0.175	0.095 (0.047)		0.035*	−0.616	0.236	0.751
reactivity	0.008	0.087	−0.225	0.096 (0.05)	−0.072	0.531	−0.579	0.618
*fish–midge* (*Crabtree*)								
maximum	0.009	0.051 (0.03)			0.121**	0.259	0.844	0.468
sensitivity	−0.018*	0.008	0.202 (0.10)*		0.048**	0.898	−0.388	0.204
reactivity	0.008	−0.116	−0.091	−0.073 (−0.04)	−0.415	−0.354	−0.367	0.859

*p < 0.05; **p < 0.01.

The canonical analysis revealed a significant and positive eigenvalue among traits for the midge pond. The eigenvector associated with the dominant eigenvalue was characterized by opposite loadings for the maximum and the sensitivity of induction, paralleling the patterns of selection indicated on the original axis (table 3). The canonical analysis for data from fish–midge pond revealed no significant eigenvalues. The first eigenvalue was linked to an eigenvector dominated by the threshold weighting, again paralleling the patterns on the original scale (table 3). The canonical analyses for each predation scenarios produced a set of three eigenvalues with different sign, suggesting that the overall selection surface is a saddle [42]. We note the absence of selection pressure on the reactivity (slope) of the response, where we also found no significant genetic variation.

### (d) The micro-evolution of a predator-induced plasticity

**Figure 3.**
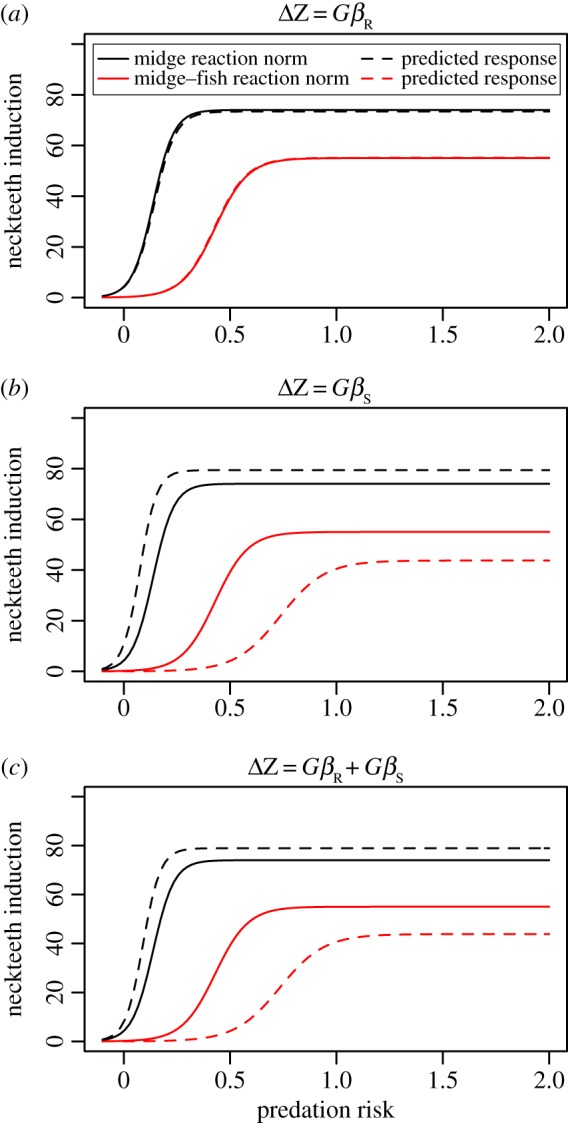
Projected neckteeth reaction norm under midge and fish predation scenarios considering the reproduction component of selection gradient (*a*), the survival component of selection gradient (*b*) and the combined reproduction and survival selection gradient (*c*). Differences in magnitude and orientation of linear selection gradient *β* and response to selection Δ*z*. (Online version in colour.)

Table 4 and figure 3 highlight the predicted response to selection of the reaction norm of induced morphological defence. Figure 3*a* captures the response of each population reaction norm assuming that only the reproduction component of the selection gradient matters. Selection driven only by reproduction drives convergence of the environment-specific reaction norms towards a maximum and sensitivity that is in between that defined by a midge or a fish–midge environment.

**Table 4.** The response to selection (Δ*Z*) using composite selection gradients based upon different weighting of reproduction (*β*_R_) and survival (*β*_S_) selection. The multivariate response to selection is partitioned into trait-specific direct (through genetic variance), indirect (through genetic covariance) and total (using all components of *G*) response in the fish and midge cue treatment.

	trait	total response	direct selection	indirect selection
*midge pond response*				
ΔZ*_β_*_(R)_	maximum	−0.089 (−0.254; −0.039)^a^	0	−0.089 (−0.254; −0.039)^a^
ΔZ*_β_*_(R)__+__0.5*β*(S)_	maximum	0.393 (0.168; 1.134)^a^	0.341 (0.146; 0.884)^a^	−0.119 (−0.045; 0.378)
ΔZ*_β_*_(R)__+_*_β_*_(S)_	maximum	0.889 (0.451; 2.292)^a^	0.680 (0.270; 1.523)^a^	0.230 (−0.039; 0.872)
ΔZ_0.5*β*(R)__+_*_β_*_(S)_	maximum	0.942 (0.478; 2.416)^a^	0.680 (0.270; 1.523)^a^	0.280 (0.017; 0.997)^a^
ΔZ*_β_*_(S)_	maximum	0.995 (0.500; 2.551)^a^	0.68 (0.27; 1.523)^a^	0.344 (0.071; 1.113)^a^
ΔZ*_β_*_(R)_	sensitivity	0.121 (0.062; 0.322)^a^	0.121 (0.062; 0.322)^a^	0
ΔZ*_β_*_(R)__+__0.5*β*(S)_	sensitivity	−0.364 (−1.099; −1.152)^a^	−0.214 (−0.627; −0.092)^a^	−0.165 (−0.598; 0.032)
ΔZ*_β_*_(R)__+_*_β_*_(S)_	sensitivity	−0.993 (−2.405; −0.404)^a^	−0.585 (−1.562; −0.301)^a^	−0.271 (−1.128; −0.037)^a^
ΔZ_0.5*β*(R)__+_*_β_*_(S)_	sensitivity	−1.222 (−2.582; −0.449)^a^	−0.645 (−1.723; −0.332)^a^	−0.271 (−1.128; −0.037)^a^
ΔZ*_β_*_(S)_	sensitivity	−1.101 (−2.772; −0.503)^a^	−0.706 (−1.885; −0.363)^a^	−0.271 (−1.128; −0.037)^a^
ΔZ*_β_*_(R)_	reactivity	0.039 (−0.013; 0.147)	0	0.039 (−0.013; 0.147)
ΔZ*_β_*_(R)__+__0.5*β*(S)_	reactivity	−0.125 (−0.420; 0.047)	0.014 (0.005; 0.237)^a^	−0.213 (−0.628; 0.05)
ΔZ*_β_*_(R)__+_*_β_*_(S)_	reactivity	−0.300 (−0.934; 0.160)	0.059 (0.006; 0.474)^a^	−0.398 (−1.388; 0.088)
ΔZ_0.5*β*(R)__+_*_β_*_(S)_	reactivity	−0.315 (−1.003; 0.166)	0.059 (0.006; 0.474)^a^	−0.384 (−1.490; 0.135)
ΔZ*_β_*_(S)_	reactivity	−0.330 (−1.080; 0.153)	0.059 (0.006; 0.474)^a^	−0.400 (−1.575; 0.125)
*fish–midge response*				
ΔZ*_β_*_(R)_	maximum	0.009 (0.004; 0.027)^a^	0	0.009 (0.004; 0.027)^a^
ΔZ*_β_*_(R)__+__0.5*β*(S)_	maximum	−0.590 (−1.587; −0.255)^a^	−0.364 (−0.884; −0.146)^a^	−0.221 (−0.754; −0.098)^a^
ΔZ*_β_*_(R)__+_*_β_*_(S)_	maximum	−1.274 (−2.974; −0.531)^a^	−0.680 (−1.523; −0.270)^a^	−0.510 (−1.459; −0.226)^a^
ΔZ_0.5*β*(R)__+_*_β_*_(S)_	maximum	−1.280 (−2.986; −0.533)^a^	−0.680 (−1.523; −0.270)^a^	−0.514 (−1.472; −0.228)^a^
ΔZ*_β_*_(S)_	maximum	−1.286 (−2.997; −0.536)^a^	−0.680 (−1.523; −0.270)^a^	−0.519 (−1.486; −0.230)^a^
ΔZ*_β_*_(R)_	sensitivity	−0.013 (−0.034; −0.007)^a^	−0.013 (−0.034; −0.007)^a^	0
ΔZ*_β_*_(R)__+__0.5*β*(S)_	sensitivity	0.646 (0.272; 1.682)^a^	0.314 (0.135; 0.919)^a^	0.229 (0.101; 0.783)^a^
ΔZ*_β_*_(R)__+_*_β_*_(S)_	sensitivity	1.348 (0.668; 3.215)^a^	0.693 (0.357; 1.850)^a^	0.519 (0.230; 1.486)^a^
ΔZ_0.5*β*(R)__+_*_β_*_(S)_	sensitivity	1.354 (0.671; 3.230)^a^	0.699 (0.360; 1.867)^a^	0.519 (0.230; 1.486)^a^
ΔZ*_β_*_(S)_	sensitivity	1.361 (0.674; 3.246)^a^	0.706 (0.363; 1.885)^a^	0.519 (0.230; 1.486)^a^
ΔZ*_β_*_(R)_	reactivity	−0.004 (−0.016; 0.001)	0	−0.004 (−0.016; 0.001)
ΔZ*_β_*_(R)__+__0.5*β*(S)_	reactivity	0.115 (−0.047; 0.75)	0	−0.115 (−0.047; 0.751)
ΔZ*_β_*_(R)__+_*_β_*_(S)_	reactivity	0.397 (−0.123; 1.561)	0	0.397 (−0.123; 1.561)
ΔZ_0.5*β*(R)__+_*_β_*_(S)_	reactivity	0.399 (−0.124; 1.568)	0	0.399 (−0.124; 1.568)
ΔZ*_β_*_(S)_	reactivity	0.400 (−0.125; 1.575)	0	0.400 (−0.125; 1.575)

^a^Posterior mode and the upper and lower bound of the 95% credibility interval are presented, and elements significantly different from zero.

Figure 3*b* shows the result of applying a hypothetical survival-only selection gradient to the components of the reaction norm. Here we see rather subtle, but theoretically expected shifts, where the maximum defence is elevated in both environments, but the midge regime sees increased sensitivity to the cue (threshold moves left), while the fish–midge regime sees decreased sensitivity (threshold moves right).

Finally, figure 3*c* shows the effect of equally weighted but combined response to selection on reproduction and survival. Here we see the consequences of combining both selection gradients and the constraint represented by the negative covariation between the maximum and the sensitivity in the *G*-matrix. The midge regime experiences a small decrease in maximum induction and less sensitivity to the cue, completely at odds with what theory might predict. By contrast, the fish–midge regime sees not only decreased sensitivity (as would be expected), but also increased maximum, which may or may not be expected (see below).

## 4. Discussion

Predator-induced defences are one of the most studied examples of phenotypic plasticity [2,18]. This research is grounded in a body of well-developed theory and empirical work spanning decades, debates and taxa [47,48] with daphnids forming a core body of this work. Empirical, evolutionary ecological research with daphnids is broad and has revealed fascinating insight into plasticity in the life history [49,50], behaviour [51,52], morphology [8,19,49] and costs of plasticity [8,26,49,53–55]. Recent work as revealed detail about *G* × *E* in, and reaction norms of, morphological and other defences [8,25,56]. Despite this breadth of work, no evidence about the potential micro-evolution of morphological reaction norms has been reported. Therefore, we explored how a predator-induced, nonlinear, reaction norm of induced morphological defence might respond evolutionarily to contrasting size-selective predation regimes.

Our method, developed in the context of *D. pulex* ecology, involved decomposing a nonlinear, predator-induced reaction norm to three biologically relevant traits. This showed that it is possible to analyse the micro-evolution of these nonlinear reaction norms with well established, character-state tools. Our data provided a fine scale description of the reaction norms and show that genetic variation might be predator-regime and population specific. Critically, by analysing the response of the reaction norm to plausible, predator-specific selection gradients, we found that a *G*-matrix, harbouring a strong covariance between the sensitivity to cues—the threshold part of this threshold trait—and the maximum response to cues, predicted a rather more uniform morphological response to predation risk than might be expected under size-selective predation theory about morphological defences [2].

Our results formalize a hypothesis that selection on survival and reproduction combine with covariance between the maximum morphological response to these cues (asymptote) and the sensitivity of morphology (threshold/inflection) to midge predation cues to shape the micro-evolutionary trajectory of predator-induced defences in *D. pulex*. This covariance between the two parts of the defence captures patterns of variation seen in figure 1 among the genotypes and is a major influence on our simulation of micro-evolutionary change.

### (a) Evolution of a reaction norm

We began our assessment of micro-evolutionary change to a nonlinear reaction norm with data showing that the maximum response (asymptote) and the sensitivity to the cue (threshold/inflection) harbour significant genetic variation (figure 1 and table 2). The data suggest that the midge population harbours little variation in sensitivity and a fair amount in the maximum induction while the fish–midge population harbours larger variation in the sensitivity, and less in the maximum. While sample sizes are low, these data provide a template on which to explore hypotheses about micro-evolutionary change in reaction norms. Importantly, we found in the combined data a negative genetic correlation between the maximum response and sensitivity to the cue, defining a biologically meaningful constraint (not trade-off) of increased sensitivity (reducing threshold) and increased induced morphology (increasing maximum). We highlight that our sigmoid model of the reaction norm specifies three potential traits, motivated by capturing variation visible in figure 1 through our experimental design with several maternal lines. While such a model has been used often in work on threshold traits, the approach and insights are not defined by the model, but by the capacity to capture meaningful levels of genetic (co)variation associated with the parameters.

We considered several ways in which selection might act on this genetic variation [41], combining empirical data on reproduction linked to variation in defence traits with size-selective theory about patterns of survival linked to the same traits. Our composite selection scenarios represent several weightings of selection linked to survival and reproduction to which a population may respond. This was combined in the multivariate breeders equation with the constraints explicit in the *G*-matrix (figure 3). Our results reveal not only the potential for substantially different response to selection pattern depending on the predation risk scenario, but also how strong covariation between traits might constrain the response.

Although our analysis is developed from data from only two populations, we argue cautiously about the effect of selection on population differentiation. However, previous evidence about cost of plasticity [8] and differences in phenotypic trajectories [25] support this possibility in relation to realistic patterns of the evolution of the reaction norm.

### (b) Covariance matters

As noted above, we found a biologically meaningful negative correlation between maximum response and sensitivity to cue that represents a potential evolutionary constraint. Were this to be an accurate representation of the three traits in a larger pool of populations, combining the selection gradients with this *G*-matrix provides an interesting perspective on how evolution might act on predator-induced plasticity. When the total response of selection is decomposed to its direct and indirect effect, the relevance of the genetic covariance became clear. The significant and highly negative genetic covariance between maximum and threshold constrained the response of selection in the two different predation scenarios. The relatively large angle between *β*'s and Δ*Z*'s reinforced this view, because the covariance resulted in a response to selection significantly different from the direction of selection. They were similar between midge (Bagshaw) and fish–midge (Crabtree) predation scenarios, reflecting the covariance pattern.

Thus, the indirect response of selection seems to play a crucial role in the total response of selection, driving similar patterns of phenotypic response from different predation scenarios [57]. If the patterns of multivariate genetic variation we find is common, the constraint may represent an important genetic pathway underpinning local adaptation in daphnids facing multiple and variable regimes of predation risk [10,26,51,58,59]. Our data suggest that the evolution of midge-induced morphological plasticity is robust to the presence of an alternate, contrasting selection pressure that does not induce the morphological change.

Although our *G*-matrix is based only on morphological trait data from only two populations, our results align with the theory about size-selective predation [20] and thus represent a plausible empirical parametrization of theory and the potential outcome of selection in natural environments. It allowed us to generate a hypothesis about ecologically driven micro-evolution of the predator-induced reaction norm of the morphological defence. It also provides a template on which one can apply theory about reaction norm evolution under more extreme selection [60].

The role of phenotypic plasticity in the process of adaption is still a topic pushing theory and discussion [27,48,61]. However, several lines of evidence suggest that phenotypic plasticity may be an important driver of evolutionary change in heterogeneous environments [4,62–64]. Crucial to these arguments is that plasticity might facilitate the maintenance of genetic variation in populations that inhabit heterogeneous environments. Phenotypic plasticity is thus a mechanism that may aid the persistence of populations, allowing for higher rates of adaptation [27,61,65–68]. Empirical evidence related to the ability of organisms to evolve in response to biotic, agonistic interactions [69–71] suggest that this link between plasticity and evolvability is real. Our data add to this knowledge, showing how fine scale, multivariate predator-induced phenotypic plasticity likely influences the outcome of evolution in heterogeneous predator environments.

## 5. Conclusion

Predation is a key selective agent in aquatic and terrestrial communities. Seasonal and spatial variation in predation risk has favoured the evolution of inducible defences, a major example of phenotypic plasticity. The response of traits to predation risk is rarely effectively characterized by a linear reaction norm describing plasticity. Characterizing genetic variation and covariation in a nonlinear relationship is not trivial, but it is vital for understanding how agents of natural selection such as predators drive the evolution of phenotypic plasticity. Using a model system of daphnids facing predation risk from multiple predators, we show, empirically, one route to describing variation in and micro-evolutionary response of a nonlinear reaction norms. Our representation of genetic (co)variance in the reaction norm allowed us to explore the potential micro-evolutionary response of the reaction norm shaped by variable predation risk and constraints on the evolution of specific features of the reaction norm.

## Supplementary Material

Supplementary Table 1

## Supplementary Material

Supplementary Table 2

## Data accessibility

Data are available from Dryad: http://dx.doi.org/10.5061/dryad.kr856 [72].

## References

[1] Lima SL 1998 Nonlethal effects in the ecology of predator–prey interactions. Bioscience 48, 25–34. (10.2307/1313225)

[2] Tollrian R, Harvell CD 1999 The ecology and evolution of inducible defenses. Princeton, NJ: Princeton University Press.

[3] Bernard MF 2004 Predator-induced phenotypic plasticity in organisms with complex life histories. Ann. Rev. Ecol. Evol. Syst. 35, 651–673. (10.2307/annurev.ecolsys.35.021004.30000024)

[4] Miner BG, Sultan SE, Morgan SG, Padilla DK, Relyea RA 2005 Ecological consequences of phenotypic plasticity. Trends Ecol. Evol. 20, 685–692. (10.1016/j.tree.2005.08.002) 16701458

[5] Pigliucci M 2005 Evolution of phenotypic plasticity: where are we going now? Trends Ecol. Evol. 20, 481–486. (10.1016/j.tree.2005.06.001) 16701424

[6] Dewitt TJ, Sih A, Wilson DS 1998 Costs and limits of phenotypic plasticity. Trends Ecol. Evol. 13, 77–81. (10.1016/S0169-5347(97)01274-3) 21238209

[7] Relyea RA 2002 Costs of phenotypic plasticity. Am. Nat. 159, 272–282. (10.1086/338540) 18707379

[8] Hammill E, Rogers A, Beckerman AP 2008 Costs, benefits and the evolution of inducible defences: a case study with *Daphnia pulex*. J. Evol. Biol. 21, 705–715. (10.1111/j.1420-9101.2008.01520.x) 18355186

[9] Tollrian R 1995 *Chaoborus crystallinus* predation on *Daphnia pulex*: can induced morphological changes balance effects of body size on vulnerability? Oecologia 101, 151–155. (10.1007/BF00317278) 28306785

[10] Boeing WJ, Ramcharan CW, Riessen HP 2006 Multiple predator defence strategies in *Daphnia pulex* and their relation to native habitat. J. Plankton Res. 28, 571–584. (10.1093/plankt/fbi142)

[11] Havel JE, Dodson SI 1987 Reproductive costs of *Chaoborus*-induced polymorphism in *Daphia pulex*. Hydrobiologia 150, 273–281. (10.1007/BF00008708)

[12] Black AR 1993 Predator-induced phenotypic plasticity in *Daphnia pulex*: life history and morphological responses to *Notonecta* and *Chaoborus*. Limnol. Oceanogr. 38, 986–996. (10.4319/lo.1993.38.5.0986)

[13] Repka S, Pihlajamaa K 1996 Predator-induced phenotypic plasticity in *Daphnia pulex*: uncoupling morphological defenses and life history shifts*. Hydrobiologia 339, 67–71. (10.1007/BF00008914)

[14] Sell AF 2000 Morphological defenses induced in situ by the invertebrate predator *Chaoborus:* comparison of responses between *Daphnia pulex* and *D. rosea*. Oecologia 125, 150–160. (10.1007/PL00008886) 28308217

[15] Lass S, Spaak P 2003 Chemically induced anti-predator defences in plankton: a review. Hydrobiologia 491, 221–239. (10.1023/A:1024487804497)

[16] Pohnert G, Steinke M, Tollrian R 2007 Chemical cues, defence metabolites and the shaping of pelagic interspecific interactions. Trends Ecol. Evol. 22, 198–204. (10.1016/j.tree.2007.01.005) 17275948

[17] Van Donk E 2007 Chemical information transfer in freshwater plankton. Ecol. Inform. 2, 112–120. (10.1016/j.ecoinf.2007.03.002)

[18] Agrawal AA 2001 Phenotypic plasticity in the interactions and evolution of species. Science 294, 321–326. (10.1126/science.1060701) 11598291

[19] Tollrian R 1995 Predator-induced morphological defenses: costs, life history shifts, and maternal effects in *Daphnia pulex*. Ecology 76, 1691–1705. (10.2307/1940703)

[20] Taylor BE, Gabriel W 1992 To grow or not to grow: optimal resource allocation for *Daphnia*. Am. Nat. 139, 248–266. (10.1086/285326)

[21] Hammill E, Kratina P, Anholt BR 2008 Non-lethal presence of predators modifies morphology and movement rates in *Euplotes*. Hydrobiologia 621, 183–189. (10.1007/s10750-008-9644-1)

[22] Nijhout HF 1999 Control mechanisms of polyphenic development in insects. Bioscience 49, 181 (10.2307/1313508)

[23] Roff DA 1996 The evolution of threshold traits in animals. Q. Rev. Biol. 71, 3–35. (10.1086/419266)

[24] Svennungsen TO, Holen ØH, Leimar O 2011 Inducible defenses: continuous reaction norms or threshold traits? Am. Nat. 178, 397–410. (10.1086/661250) 21828995

[25] Dennis SR, Carter MJ, Hentley WT, Beckerman AP 2011 Phenotypic convergence along a gradient of predation risk. Proc. R. Soc. B 278, 1687–1696. (10.1098/rspb.2010.1989) PMC3081771 21084350

[26] Parejko K, Dodson SI 1991 The evolutionary ecology of an antipredator reaction norm: *Daphnia pulex* and *Chaoborus americanus*. Evolution 45, 1665–1674. (10.2307/2409787) 28564128

[27] Lind MI, Yarlett K, Reger J, Carter MJ, Beckerman AP 2015 The alignment between phenotypic plasticity, the major axis of genetic variation and the response to selection. Proc. R. Soc. B 282, 20151651 (10.1098/rspb.2015.1651) PMC4614775 26423845

[28] Schluter D 1988 Estimating the form of natural selection on a quantitative trait. Evolution 42, 849–861. (10.2307/2408904) 28581168

[29] Blows MW, Brooks R, Kraft PG 2003 Exploring complex fitness surfaces: multiple ornamentation and polymorphism in male guppies. Evolution 57, 1622–1630. (10.1111/j.0014-3820.2003.tb00369.x) 12940366

[30] Carpenter SR et al. 1987 Regulation of lake primary productivity by food web structure. Ecology 68, 1863–1876. (10.2307/1939878) 29357166

[31] Reger J 2013 The quantitative genetic basis of inducible defences and life-history plasticity in *Daphnia pulex*. PhD thesis, University of Sheffield.

[32] ASTM. 1989 Standard guide for conducting acute toxicity test with fishes, macroinvertebrates and amphibians. In Annual book of ASTM standards, pp. 379–397. West Conshohocken, PA: ASTM International.

[33] R Development Core Team. 2011 R: a language and environment for statistical computing. Vienna, Austria: R Foundation for Statistical Computing.

[34] Lampert W, Trubetskova I 1996 Juvenile growth rate as a measure of fitness in daphnia. Funct. Ecol. 10, 631–635. (10.2307/2390173)

[35] Hadfield JD 2010 MCMC methods for multi-response generalized linear mixed models: the MCMCglmm R package. J. Stat. Softw. 33, 1–22. (10.18637/jss.v033.i02)20808728

[36] Lande R, Arnold SJ 1983 The measurement of selection on correlated characters. Evolution 37, 1210–1226. (10.2307/2408842) 28556011

[37] Lenth RV 2009 Response-surface methods in R, using rsm. J. Stat. Softw. 32, 1–17. (10.18637/jss.v032.i07)

[38] Brooks R, Hunt J, Blows MW, Smith MJ, Bussière LF, Jennions MD 2005 Experimental evidence for multivariate stabilizing sexual selection. Evolution 59, 871–880. (10.1111/j.0014-3820.2005.tb01760.x) 15926696

[39] Revell LJ, Mahler DL, Sweeney JR, Sobotka M, Fancher VE, Losos JB 2010 Nonlinear selection and the evolution of variances and covariances for continuous characters in an anole. J. Evol. Biol. 23, 407–421. (10.1111/j.1420-9101.2009.01911.x) 20039998

[40] Manly BFJ 1997 RT. *A program for randomization testing,* version 2.0. Otago, New Zealand: Centre for Applications of Statistics and Mathematics. University of Otago.

[41] Stinchcombe JR, Agrawal AF, Hohenlohe PA, Arnold SJ, Blows MW 2008 Estimating nonlinear selection gradients using quadratic regression coefficients: double or nothing? Evolution 62, 2435–2440. (10.1111/j.1558-5646.2008.00449.x) 18616573

[42] Blows MW, Brooks R 2003 Measuring nonlinear selection. Am. Nat. 162, 815–820. (10.1086/378905) 14737718

[43] Spitze K 1991 Chaoborus predation and life-history evolution in *Daphnia pulex*: temporal pattern of population diversity, fitness, and mean life history. Evolution 45, 82–92. (10.2307/2409484) 28564082

[44] Pastorok RA 1981 Prey vulnerability and size selection by *Chaoborus* larvae. Ecology 62, 1311–1324. (10.2307/1937295)

[45] Brooks JL, Dodson SI 1965 Predation, body size, and composition of plankton. Science 150, 28–35. (10.1126/science.150.3692.28) 17829740

[46] Lande R 1979 Quantitative genetic analysis of multivariate evolution, applied to brain: body size allometry. Evolution 33, 402–416. (10.2307/2407630) 28568194

[47] Laforsch C, Ngwa W, Grill W, Tollrian R 2004 An acoustic microscopy technique reveals hidden morphological defenses in *Daphnia*. Proc. Natl Acad. Sci. USA 101, 15 911–15 914. (10.1073/pnas.0404860101) PMC528736 15520396

[48] Via S, Gomulkiewicz R, De Jong G, Scheiner SM, Schlichting CD, Van Tienderen PH 1995 Adaptative phenotypic plasticity: concensus and controversy. Trends Ecol. Evol. 10, 212–217. (10.1016/S0169-5347(00)89061-8) 21237012

[49] Boersma M, Spaak P, De Meester L 1998 Predator-mediated plasticity in morphology, life history, and behavior of *Daphnia:* the uncoupling of responses. Am. Nat. 152, 237–248. (10.1086/286164) 18811388

[50] Spitze K, Burnson J, Lynch M 1991 The covariance structure of life-history characters in *Daphnia pulex*. Evolution 45, 1081–1090. (10.2307/2409717) 28564186

[51] De Meester L 1993 Genotype, fish-mediated chemical, and phototactic behavior in *Daphnia magna*. Ecology 74, 1467–1474. (10.2307/1940075)

[52] De Meester L 1989 An estimation of the heritability of phototaxis in *Daphnia magna* straus. Oecologia 78, 142–144. (10.1007/BF00377210) 28311914

[53] Scheiner SM, Berrigan D 1998 The genetics of phenotypic plasticity. VIII. The cost of plasticity in *Daphnia pulex*. Evolution 52, 368–378. (10.2307/2411074) 28568340

[54] Spitze K, Sadler TD 1996 Evolution of a generalist genotype: multivariate analysis of the adaptiveness of phenotypic plasticity. Am. Nat. 148, S108–S123. (10.1086/285905)

[55] Stibor H, Navarra DM 2000 Constraints on the plasticity of *Daphnia magna* influenced by fish-kairomones. Funct. Ecol. 14, 455–459. (10.1046/j.1365-2435.2000.00441.x)

[56] Beckerman AP, Rodgers GM, Dennis SR 2010 The reaction norm of size and age at maturity under multiple predator risk. J. Anim. Ecol. 79, 1069–1076. (10.1111/j.1365-2656.2010.01703.x) 20522144

[57] Kingsolver JG, Gomulkiewicz R, Carter PA 2001 Variation, selection and evolution of function-valued traits. Genetica 112–113, 87–104. (10.1023/A:1013323318612) 11838789

[58] Boersma M, De Meester L, Spaak P 1999 Environmental stress and local adaptation in *Daphnia magna*. Limnol. Oceanogr. 44, 393–402. (10.4319/lo.1999.44.2.0393)

[59] De Meester L 1996 Evolutionary potential and local genetic differentiation in a phenotypically plastic of a cyclical parthenogen, *Daphnia magna*. Evolution 50, 1293–1298. (10.1111/j.1558-5646.1996.tb02369.x) 28565281

[60] Chevin L-M, Lande R, Mace GM 2010 Adaptation, plasticity, and extinction in a changing environment: towards a predictive theory. PLoS Biol. 8, e1000357 (10.1371/journal.pbio.1000357) 20463950 PMC2864732

[61] Ghalambor CK, McKay JK, Carroll SP, Reznick DN 2007 Adaptive versus non-adaptive phenotypic plasticity and the potential for contemporary adaptation in new environments. Funct. Ecol. 21, 394–407. (10.1111/j.1365-2435.2007.01283.x)

[62] Dewitt TJ 1998 Costs and limits of phenotypic plasticity: tests with predator-induced morphology and life history in a freshwater snail. J. Evol. Biol. 11, 465–480. (10.1007/s000360050100)

[63] Pfennig DW, Wund MA, Snell-Rood EC, Cruickshank T, Schlichting CD, Moczek AP 2010 Phenotypic plasticity's impacts on diversification and speciation. Trends Ecol. Evol. 25, 459–467. (10.1016/j.tree.2010.05.006) 20557976

[64] Thibert-Plante X, Hendry AP 2011 The consequences of phenotypic plasticity for ecological speciation. J. Evol. Biol. 24, 326–342. (10.1111/j.1420-9101.2010.02169.x) 21091567

[65] Burger R, Lynch M 1995 Evolution and extinction in a changing environment: a quantitative-genetic analysis. Evolution 49, 151–163. (10.2307/2410301) 28593664

[66] Gomulkiewicz R, Holt RD 1995 When does evolution by natural selection prevent extinction? Evolution 49, 201–207. (10.2307/2410305) 28593677

[67] Draghi JA, Whitlock MC 2012 Phenotypic plasticity facilitates mutational variance, genetic variance, and evolvability along the major axis of environmental variation. Evolution 66, 2891–2902. (10.1111/j.1558-5646.2012.01649.x) 22946810

[68] Lande R, Shannon S 1996 The role of genetic variation in adaptation and population persistence in a changing environment. Evolution 50, 434–437. (10.2307/2410812) 28568879

[69] Carroll SP, Hendry AP, Reznick DN, Fox CW 2007 Evolution on ecological time-scales. Funct. Ecol. 21, 387–393. (10.1111/j.1365-2435.2007.01289.x)

[70] Gilchrist GW, Lee CE 2007 All stressed out and nowhere to go: does evolvability limit adaptation in invasive species? Genetica 129, 127–132. (10.1007/s10709-006-9009-5) 16924404

[71] Yoshida T, Jones LE, Ellner SP, Fussmann GF, Hairston NG Jr 2003 Rapid evolution drives ecological dynamics in a predator–prey system. Nature 424, 303–306. (10.1038/nature01767) 12867979

[72] Carter MJ, Lind MI, Dennis SR, Hentley W, Beckerman AP 2017 Data from: Evolution of a predator-induced, non-linear reaction norm. *Dryad Digital Repository*. (10.5061/dryad.kr856) PMC5577476 28835554

