# Supplementary material for: Evolution of a predator-induced, nonlinear reaction norm
Source: Proc Biol Sci. 2017 Aug 23;284(1861):20170859. doi: 10.1098/rspb.2017.0859 (PMC5577476; doi:10.1098/rspb.2017.0859)
Supplement: Supplementary Table 1 [file rspb20170859supp1.docx]

**Supplementary Table 1**. The trait-specific coefficients for the composite selection gradients.

Each gradient is standardized to a length of 1.

|  | Trait | β_R_ | β_R_+0.5β_S_ | β_R_+β_S_ | 0.5β_R_+β_S_ | β_S_ |
| --- | --- | --- | --- | --- | --- | --- |
| Midge pond | Maximum | 0.000 | 0.5 | 1 | 1 | 1 |
|  | Sensitivity | 0.171 | -0.329 | -0.829 | -0.914 | -1 |
|  | Reactivity | 0.000 | 0.5 | 1 | 1 | 1 |
|  |  |  |  |  |  |  |
| Fish-Midge Pond | Maximum | 0.000 | 0.5 | -0.707 | 1 | -1 |
|  | Sensitivity | -0.018 | -0.518 | 0.688 | -1.01 | 1 |
|  | Reactivity | 0.000 | 0.5 | 0.000 | 1 | 0.000 |
