# Supplementary material for: Evolution of a predator-induced, nonlinear reaction norm
Source: Proc Biol Sci. 2017 Aug 23;284(1861):20170859. doi: 10.1098/rspb.2017.0859 (PMC5577476; doi:10.1098/rspb.2017.0859)
Supplement: Supplementary Table 2 [file rspb20170859supp2.docx]

- **Supplementary Table 2**. Model comparisons using Deviance Information Criterion (DIC). This method is suggested for Hadfield 2010 to determine the significant estimates from MCMC procedure.

| - Pooled population - (Bagshaw + Crabtree) | - DIC - Genetic + Residual | - DIC - Residual |  |
| --- | --- | --- | --- |
| - *Variance* |  |  |  |
| - Maximum | - 53.37 | - 77.01 |  |
| Sensitivity | - 47.02 | - 88.42 |  |
| Reactivity | - 107.82 | - 105.96 |  |
| - *Covariances* |  |  |  |
| - *Trivariate model* - (Max-Sens-Reac) | - 195.92 | - 207.46 |  |
